# Chronic Total Occlusion Percutaneous Coronary Intervention in Patients With Prior Coronary Artery Bypass Graft: Current Evidence and Future Perspectives

**DOI:** 10.3389/fcvm.2022.753250

**Published:** 2022-04-11

**Authors:** Lei Guo, Haichen Lv, Xiaomeng Yin

**Affiliations:** Department of Cardiology, The First Affiliated Hospital of Dalian Medical University, Dalian, China

**Keywords:** coronary chronic total occlusions, percutaneous coronary intervention, prior coronary artery bypass graft, characteristics, success rates, complications, outcomes

## Abstract

Coronary chronic total occlusion (CTO), which occurs in 18. 4–52% of all patients referred for coronary angiography, represents one of the last barriers in coronary intervention. Approximately half of all patients with prior coronary artery bypass graft (CABG), who undergo coronary angiography, are diagnosed with coronary CTO. In fact, these patients often develop recurrent symptoms and events, necessitating revascularization. Currently, there is neither a consensus nor developed guidelines for the treatment of CTO patients with prior CABG, and the prognosis of these patients remains unknown. In this review, we discuss current evidence and future perspectives on CTO revascularization in patients with prior CABG, with special emphasis on clinical and lesion characteristics, procedural success rates, periprocedural complications, and long-term outcomes.

## Introduction

Coronary chronic total occlusion (CTO), which widely occurs in patients who undergo routine invasive coronary angiography with an incidence rate of 18.4–52%, represents one of the last frontiers of coronary interventions (1–4). Successful CTO percutaneous coronary intervention (PCI) has been associated with improved long-term survival, left ventricular function and quality of life, as well as reduced need for coronary artery bypass graft (CABG) surgery (5–10). Despite the significant role played by this strategy in lowering prevalence of adverse events and enhancing outcomes, over the past decades, patients with CTO were often managed conservatively or surgically, rather than with PCI. In fact, according to the National Cardiovascular Disease Registry (NCDR) CathPCI registry, CTO PCI only represents 3.8% of the total 594,510 PCI cases for stable coronary artery disease (CAD) (11). However, recent technological advancements and intervention strategies have contributed to the higher initial success rates and acceptable complication rates at experienced centers (12, 13), and these advancements have increased interest in application of CTO PCI in patients with appropriate indications (14).

Previous studies have shown that CABG has been widely used for treatment of patients with multivessel CAD and left main disease, and proven to significantly improve their long-term clinical outcomes (15, 16). A recent study showed that 54% of patients with prior CABG who underwent coronary angiography were diagnosed with CTO, owing to the fact that coronary bypass is associated with accelerated progression of atherosclerosis of native coronary arteries (1). Notably, patients with saphenous vein graft (SVG) often develop recurrent ischemic symptoms, which necessitates revascularization in this group of patients (17). To date, however, neither recognized guidelines nor accepted consensus have been developed targeting treatment of this group of patients, and the prognosis of these patients remains unknown. Here, we discuss current progress and future perspectives of CTO revascularization in patients with prior CABG, focusing on clinical and lesion characteristics, procedural success rates, periprocedural complications, and long-term outcomes.

## Bypass Graft PCI is Avoided in Patients With Prior CABG

Failure of bypass graft or progression of native CAD implies that patients with prior CABG often require additional revascularization, which commonly involves right coronary artery (RCA) or left circumflex coronary artery but less often the left anterior descending (LAD) artery (18). Notably, graft failure, especially for SVGs, can occur early (after CABG surgery) or late (after several months or years following surgical revascularization). Previous studies have reported that 40% of SVGs will be occluded at 1 year, and 50% of SVGs will be diseased or occluded during the first 10 years of follow-up (19–21). Early post-operative graft failure is mainly caused by conduit defects, poor native vessel runoff and anastomotic technical errors, or competitive flow with the native coronary arteries (22). One month after CABG surgery, SVG disease development starts with neointimal hyperplasia, followed by proliferation and migration of smooth muscle cells with deposition of extracellular matrix, which results in luminal loss (23), and the progression of the atherosclerotic plaque leads to bypass graft stenosis or occlusion. There is a remodeling process of SVGs after surgery. During this process, pro-inflammatory factors, cytokines in arterial wall and atherogenic lipoproteins in plasma cause formation of a highly atherogenic substrate, on which atherosclerosis develops (24, 25).

Furthermore, SVG lesions are often degenerated, and are prone to distal embolization and high restenosis. SVG PCI was associated with higher risk of no-reflow and periprocedural myocardial infarction (MI) (26, 27). Accumulating evidences have suggested that embolization of atheromatous material to the distal vasculature, coupled with severe vasospasm induced by microembolization of platelet-rich thrombi that release vasoactive agents resulting in microvascular obstruction, are the possible mechanisms of no reflow (26, 28). To minimize the chance of distal embolization and prevent reflow, several strategies, such as administration of vasodilators, embolic protection devices, direct stenting, and use of undersized stents can be applied (29, 30).

A previous meta-analysis of 6 randomized clinical trials, comprising 1,582 patients, demonstrated that high incidence of procedural complications, such as suture dehiscence and perforation, as well as short and long-term major adverse events, including a 2-fold rate of in-hospital deaths, were more common in bypass graft PCI compared to native coronary PCI (26, 31). The ongoing PROCTOR (Percutaneous Coronary Intervention of Native Coronary Artery vs. Venous Bypass Graft in Patients with Prior Coronary Artery Bypass Graft Surgery) Trial, which plan to enroll 584 patients with a clinical indication for PCI and a dysfunctional graft on the target vesselional venous bypass graft with 3 years follow-up, may give more evidences to us.

Previous study reported that SVG PCIs account for approximately 6% of all PCIs performed in the United States (27). The guidelines of the American College of Cardiology/American Heart Association recommend that class III for PCI of SVG CTOs, and SVG CTOs should generally not be recanalized, due to a high risk of restenosis (32). Similarly, the 2018 ESC/EACTS guidelines for myocardial revascularization recommend that PCI should be considered in the native vessel rather than in an SVG graft (Class IIa, Level of Evidence: C) (33). Notably, these patients are more likely to be predisposed to a higher surgical risk, such as acute coronary syndrome (ACS), serious comorbidities and frailty, which are contraindications to the use of extracorporeal circulation. However, PCI is a safe and effective approach, hence suitable for this group of patients. Furthermore, due to increased age, frailty and multiple comorbid illnesses, repeat CABG has been associated with limited symptomatic improvement, and more adverse events (2 to 4-fold mortality), compared with initial CABG mainly driven by comorbidity (34, 35).

In summary, 50% of SVGs will be diseased or occluded during the first 10 years after CABG surgery. However, SVG PCI typically carries a higher risk of procedural complications, as well as short and long-term major adverse events. Previous studies and guidelines showed, in this case, CTO PCI, rather than CABG, is recommended for revascularization. Therefore, performing CTO PCI in native coronary artery guarantees more favorable outcomes in CTO patients with prior CABG who develop recurrent symptoms.

## Characteristics and CTO PCI Success Rates in Patients With Prior CABG

CTO registries indicated that prior CABG is a predictor of procedural failure, and is more frequent in patients with failed CTO PCI procedures (5, 36). A recent study from the REgistry of Crossboss and Hybrid procedures in FrAnce the NetheRlands, BelGium and UnitEd Kingdom (RECHARGE) cohort found a significantly lower success rate (71.9%) in the post-CABG group, relative to no-CABG group (88.7%, *p* < 0.001) (37). Furthermore, Michael et al. (38) analyzed data for 1,363 subjects from the Multicenter US Registry and found similar results among patients with prior CABG. The low technical success rates of CTO PCI in patients with prior CABG may reflect the enormous difficulty of intervention in this population. We attribute this phenomenon to the following reasons: Firstly, when compared to patients without prior CABG, those with prior CABG who underwent CTO revascularization were older, and exhibited more comorbidities, including hypertension, diabetes, prior MI, previous stroke, chronic kidney insufficiency and left ventricular dysfunction (37, 39–41), which have previously been shown to be independent predictors of CTO PCI failure (11, 42). Secondly, regarding lesion characteristics, patients with prior CABG who underwent CTO intervention often exhibited higher complexity of the CTO lesion and vessel anatomy, mainly because CABG can accelerate development of native coronary artery atherosclerosis due to the competitive flow generated by the grafting process (43). In addition, sternal reentry, pericardial adhesions, *in situ* arterial grafts, and patent but diseased SVGs all increase the complexity and risk of coronary reoperations. Notably, the above two were also the main reasons why patients with prior CABG were not eligible for redo CABG, according to a study from the Cleveland Clinic (44).

Sakakura et al. (45) reported that CTOs in patients with prior CABG manifested pathological features of accelerated atherosclerosis progression, including moderate/severe calcification, moderate negative remodeling, and more blunt stumps than those without CABG. It is possible that these differences in pathology may negatively impact the success rates of CTO PCI in such patients. In fact, results from a recent meta-analysis comprising 8,131 patients who underwent CTO PCI, of which 2,163 had prior while 5,968 were without CABG, revealed that patients with prior CABG had more calcified and longer lesions, and higher Japanese-chronic total occlusion (J-CTO) score (i.e. more complex lesions) relative to those without prior CABG. Moreover, prior CABG has been associated with longer CTO durations as well as more pronounced calcification, blunt proximal cap and vessel tortuosity, due to the shrinkage of the occluded bypass graft or vessel distortion at the time of bypass grafting (37), which elevate the technical difficulty. The underlying mechanism of native arterial calcification has largely been attributed to blood stasis and low shear stress resulting from competitive flow between the native and bypass graft (46, 47). In addition, PCI on calcified lesion represents a challenge for the interventionalist, and has been associated with lower procedural success rates, relatively higher incidence of procedural complications and increased rates of restenosis, due to insufficient stent expansion (48). Besides, CABG surgery causes distortion, displacement, and deformation of the native coronary arteries, thereby hindering CTO crossing attempts, and making CTO PCI more technically challenging. Additionally, complications during CTO PCI procedures, such as coronary perforation, might negatively impact its success rate (49).

Notably, previous researchers have frequently performed a retrograde approach in CTO patients with prior CABG (38, 41, 50). For example, a previous meta-analysis reported 34.7 and 21.9% success rates in patients with and without prior CABG (*p* < 0.001), respectively (51). The wide adoption of the retrograde approach in these patients was likely related to complexity of the CTO lesion, which requires application of multiple crossing techniques. Bypass grafts, both SVG and left internal mammary artery (LIMA), can serve as retrograde conduits. For example, Xenogiannis et al. (52) compared retrograde cases via SVGs with other collateral vessels, and found that the former was associated with significantly higher rates of technical (85 vs. 78%; *p* = 0.04) and procedural success (81 vs. 74%; *p* = 0.04) than the latter. On the other hand, Dautov et al. (53) examined the feasibility and safety of CTO PCI via SVGs compared to collateral channels or an antegrade-only approach in patients with prior CABG, and found that retrograde cases via SVGs were safe and effective. Notably, use of SVG reduced radiation, contrast volume, fluoroscopic and procedural time, and was further associated with an equally high success and low complications (53). Based on these findings, retrograde approach is recommended for native artery CTO PCI via an occluded or for patients with SVG when the anatomy suggests that the retrograde approach would be more effective. The LIMA is not frequently applied in CTO PCI practice (2%), possibly due to performance of redo CABG in cases of LIMA failure. Besides, the hazard associated with that approach (LIMA is used as a retrograde conduit) should be considered. For example, if an attempt is made to access distal LAD septal collaterals to open a RCA via the LIMA, the risk of kinking the LIMA and inducing ischemia and shock is significant (54). Consequently, this approach should only be used as the last resort. Conversely, retrograde CTO PCI may be safer in patients with prior CABG, because pericardial adhesions may reduce the likelihood of tamponade in CABG case of collateral vessel perforation (37, 51). On the other hand, it should be noticed that if a retrograde approach is attempted, the operator will be forced to use the microchannel via the bypass graft, which may be linked to more complex procedures and a wider area of ischemia (55, 56).

Even though the success rate of CTO-PCI in patients with prior CABG was significantly lower than that in the those without prior CABG, the recent technological advancements and development of novel targeted devices, have made the CTO intervention safe and effective (57). Previous studies showed that prior CABG patients more often had dual injection (71–77%) and femoral access (74–88%) (39, 50). CTO PCIs in prior CABG patients more often required use of antegrade dissection/re-entry (ADR) (35%) and the retrograde (42–53%), whereas the antegrade wire escalation was used less frequently (39, 50). Furthermore, application of the “hybrid approach”, especially the retrograde approach via SVGs, has significantly improved the resulting technical success, from 79.7 to 88.1%, in prior CABG patients (39).

**Figure 1:**
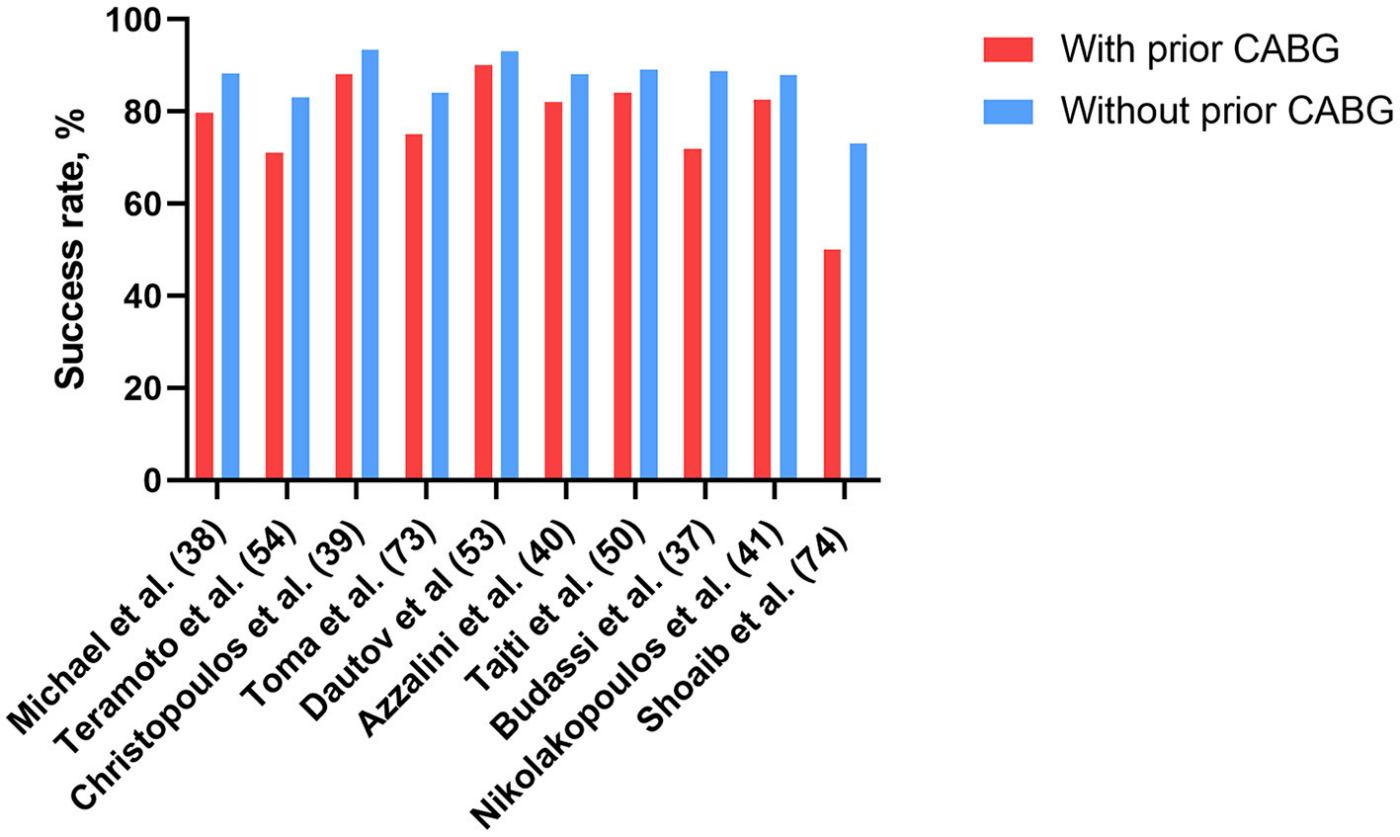
Successful rates of CTO-PCI in patients with and without prior CABG in recent studies. CABG, coronary artery bypass grafting; CTO, chronic total occlusion; PCI, percutaneous coronary intervention.

Overall, although the relatively low technical success rates of CTO PCI in patients with prior CABG due to worse baseline risk profiles and higher complexity of the CTO lesion, ADR, retrograde approach via SVGs, even hybrid approach, coupled with a growing operator experience, maintain high success rates. These have enhanced the interest and confidence for application of CTO PCI in these high-risk patients. A summary of recent studies that have evaluated CTO PCI in patients with prior CABG is provided in Table 1, and the corresponding success rates are presented in Figure 1.

**Table 1:** Recent studies of CTO-PCI in patients with prior CABG.

**Study**	**No. of patients**	**J-CTO score**	**Retrograde approach, %**	**Technical success, %**	**Procedural success, %**	**Procedural complications and in-hospital outcomes** **pCABG vs. nCABG**
Michael et al. (38)						2.1 vs. 1.5%, *p =* 0.392
pCABG nCABG	508 855	NA NA	46.7 27.1	79.7 88.3	78.1 87.2	PCABG: death (perforation or intracranial bleeding) (*n =* 2), perforation (*n =* 2), donor vessel dissection (*n =* 1), MI (*n =* 4); nCABG: death (tamponade) (*n =* 1), perforations with tamponade (*n =* 8), donor vessel dissection (*n =* 1), MI (*n =* 1), stent thrombosis (*n =* 1)
Teramoto et al. (54)						Distal embolization (1.4 vs. 3.2%, *P =* 0. 0.17)
pCABG	153	NA	47	71	NA	type A coronary perforation (15.5 vs. 14.4%, *p =* 0.02)
nCABG	1,139	NA	37	83	NA	
Christopoulos et al. (39)						1.1 vs. 2.1%, *p =* 0.40
pCABG nCABG	176 320	3.12 ± 1.03 2.41 ± 1.21	39 24	88.1 93.4	87.5 92.5	PCABG: death (vascular access complication) (*n =* 1), MI (*n =* 1); nCABG: death (cardiac tamponade) (*n =* 1), MI (*n =* 4), emergency PCI (*n =* 1), tamponade with pericardiocentesis (*n =* 2)
Toma et al. (58)						Vascular access complication (0.7 vs. 0.4%), perforation (1.0 vs. 0.1%)
pCABG nCABG	292 1,710	NA NA	42 21	NA NA	75 84	cardiac tamponade (0.7 vs. 0.5%), bleeding requiring transfusion RBC (0.7 vs. 0.6%), stroke (0.3 vs. 0.1%)
Dautov et al. (53)						Death (1.1 vs. 0.3%), MI (3.4 vs. 1.4%), tamponade (0.6 vs. 1.4%)
pCABG nCABG	175 295	2.5 ± 1.3 2.1 ± 1.2	57 48	90 93	NA NA	major bleeding (0.6 vs. 1.0%), vascular complication (0.6 vs. 0%), CIN (4.6 vs. 1.0%, *p =* 0.02)
Azzalini et al. (40)						3.7 vs. 1.5%, *P =* 0.004
pCABG nCABG	401 1,657	2.3 ± 1.2 1.7 ± 1.2	40 22	82 88	81 87	Death (0.8 vs. 0.1%; *P =* 0.005), perforation (12.0 vs. 5.2%, *P* < 0.001), MI (2.0% vs. 0.5%, *P =* 0.002), tamponade (0.2 vs. 0.6%), vascular complication (1.0 vs. 1.1%), major bleeding
						(1.0 vs. 0.7%), CIN (0.7 vs. 0.2%), stroke (0.7 vs. 0.3%)
Tajti et al. (50)						Death (1.1 vs. 0.4%, *p =* 0.016), MI (1.5 vs. 0.8%), stroke (0.2 vs.
pCABG	1,101	2.9 ± 1.2	53	84	82	0.3%), perforation (7.1 vs. 3.1%, *p* < 0.001), cardiac tamponade (0.1
nCABG	2,317	2.2 ± 1.3	30	89	87	vs. 1.0%, *p =* 0.002), pericardiocentesis (0 vs. 1.3%, *p* < 0.001), re-PCI (0.5 vs. 0.3%), re-CABG (0.2 vs. 0.1%)
Budassi et al. (37)						Death (0 vs. 0.3%, *p =* 1), stroke (0.5 vs. 0.2%, *p =* 0.4), MI (4.1 vs.
pCABG	217	2.9 ± 1.2	58.5	71.9	NA	0.7%, *p =* 0.04), major bleeding (1.8 vs. 1.9%, *p =* 1), (1.1 vs. 0.4%
nCABG	1,035	2.1 ± 1.2	28.4	88.7	NA	*p =* 0.016), access site bleeding (1.4 vs. 0.3%, *p =* 0.07), cardiac tamponade (0.5 vs. 1.4%, *p =* 0.33), acute renal failure (0 vs. 0.2%, *p =* 0.99)
Nikolakopoulos et al. (41)						Pericardiocentesis (0 vs. 1.3%, *p =* 0.01), stent thrombosis (0.2 vs. 0.5%, *p =* 0.40)
pCABG	498	2.9 ± 1.1	47.4	82.6	82	In-hospital outcomes: MACE (3.4 vs. 3%, *p =* 0.65), death (2.4 vs. 1%
nCABG	1,074	2.2 ± 1.3	28.2	87.9	86	*p =* 0.04), MI (1 vs. 0.6%, *p =* 0.33), stroke (0.2 vs. 0.2%, *p =* 0.94), re-PCI (0 vs. 0.2%, *p =* 0.21)
Shoaib et al. (59)						Procedural complications (9 vs. 8%, *p =* 0.81)
pCABG	3,233	NA	NA	50	NA	In-hospital outcomes: MACE (1.1 vs. 0.91%, *p =* 0.95), death (0.34 vs.
nCABG	16,848	NA	NA	73	NA	0.18%, *p =* 0.44), stroke (0 vs. 0.04%, *p =* 0.94), major bleeding (1.19 vs. 1.14%, *p =* 0.21)

*CABG, coronary artery bypass grafting; CIN, contrast-induced nephropathy; CTO, chronic total occlusion; J-CTO, Japanese-chronic total occlusion; MACE, major adverse cardiovascular events; MI, myocardial infarction; NA, not applicable; nCABG, no-CABG; PCI, percutaneous coronary intervention; pCABG, post-CABG; RBC, red blood cells*.

## Procedural Complications and In-Hospital Outcomes in Patients With Prior CABG

Previous studies have demonstrated that total operating and fluoroscopy times, as well as air kerma radiation doses and volumes of contrast agent administered are higher in CTO patients with prior CABG relative to those without, and these have been attributed to the complexity of CTO lesion (38, 51). Consequently, these patients are predisposed to a high risk for contrast-induced nephropathy (CIN), hemodialysis and dermatitis (37, 38, 53, 60). Dautov et al. (53) found that CTO patients with prior CABG who underwent PCI had approximately 4.6% incidence of CIN, which was significantly higher than that in those without prior CABG (4.6 vs. 1.0%, *p* = 0.02). Previous studies have also shown that pre-procedural hydration, limiting contrast volume [to < 3.7 × the patients' creatinine clearance is recommended (61)], minimizing the frequency of test injections, and aborting the procedure in cases where CTO crossing has not been achieved before reaching a pre-determined contrast volume limit, as well as using iso-osmolar contrast media, intravascular ultrasound or non-contrast-based optical coherence tomography, microcatheter tip-injections instead of injections via the guiding catheter, may lower the risk for CIN (62–65). In fact, reducing exposure to radiation can be accomplished in several ways, such as using lower frame rate fluoroscopy, limiting the use of cine angiography and using the “fluoro-store” function instead, as well as frequently changing imaging angles. Usually, CTO PCI is stopped after 6–8 Gy air kerma radiation dose is reached, without successful lesion crossing, which is similar to contrast volume administration (61, 64). Additionally, using 7.5 frame per second fluoroscopy, coupled with shielding during CTO PCI can achieve similar effect compared with non-CTO PCI (66).

Notably, coronary perforation is a common complication during CTO PCI procedures in these patients, owing to the complex anatomy of lesions. Previous studies have demonstrated that CTO PCI in patients with prior CABG was associated with a high rate of coronary perforation (6.9–12%) (40, 50), consistent with the report of Megaly et al. (51), who reported comparable results (7.3 vs. 4.9%; odds ratio (OR): 2.07 [95% confidence interval (CI): 1.49–2.86]; *p* < 0.001). Results of a study from the British cardiovascular intervention society database showed that CTO intervention was an independent factor for perforation (67), and Azzalini et al. (49) found that patients with coronary perforation exhibited higher J-CTO scores, more often required the retrograde approach, and had lower success rates. Although the retrograde approach has been frequently applied in CTO patients with prior CABG, it has been associated with a higher risk of perforation relative to the antegrade approach (50). A recent study also found that a heavier burden of calcification might contribute to the elevated perforation rate (68). Both guidewire passage via the tiny collateral channels during the retrograde approach and result in collateral channel damage, and the aggressive balloon dilation in severely atherosclerotic vessels contributed to the perforation in such high-risk population. According to a recently published global expert consensus for CTO PCI, specific expertise and volume, as well as availability of dedicated equipment, are required to facilitate prevention and management of coronary perforation (69). Notably, covered stents, as effective devices for coronary perforation, are stiff devices that are difficult to navigate through tortuous vessels of CTO lesion in prior CABG patients, suggesting that the operation process may exacerbate the risk of periprocedural complications, including longer procedure time and fluoroscopy time, higher air kerma radiation dose, and larger contrast volume (46). Interestingly, the rate of coronary perforation among these patients is high, whereas the rate of pericardial tamponade is low (0–0.2%) with lesser pericardiocentesis (40, 50). A possible explanation for this phenomenon is existence of potential protective effect of pericardial adhesion in patients with previous CABG, where less free space is evident in the pericardial cavity, which subsequently reduces the risk of cardiac tamponade. However, pericardial effusions and tamponade can occur, and these events can be lethal during CTO PCI in patients with prior CABG (70). According to the OPEN-CTO registry, 4 perforations led to death of 365 patients with prior CABG (1.1%) (71). Therefore, immediate surgery or computed tomography-guided drainage is required for effective treatment when tamponade occurs, because pericardial tamponade may develop loculated hematomas that can compress the atria or the ventricles, potentially progressing to cardiogenic shock in these patients (72).

Although numerous studies have investigated in-hospital outcomes of CTO PCI in patients with prior CABG, the results are inconsistent (37, 41, 50, 54). For example, Megaly et al. (51) performed a meta-analysis, comprising 8,131 patients and found that patients with prior CABG exhibited a higher incidence of in-hospital mortality (0.8 vs. 0.3%; OR: 2.77 [95% CI: 1.43–5.39]; *p* = 0.003), and MI (1.4 vs. 0.5%; OR: 2.46 [95% CI: 1.46–4.15]; *p* < 0.001), compared with those without prior CABG. These results were consistent with the findings of Liu et al. (73). However, both groups exhibited similarities with regards to major bleeding (OR, 1.51; 95% CI, 0.90–2.53; *p* = 0.11), acute cerebrovascular events (0.3 vs. 0.3%; OR: 1.51 [95% CI: 0.49–4.66]; p = 0.47), vascular access complication (OR: 1.50; 95% CI: 0.93–2.41; *p* = 0.10), and emergency CABG (OR: 0.99; 95% CI: 0.25–3.91; *p* = 0.99) (51, 73).

Overall, patients with prior CABG exhibit a higher incidence of procedural complications and in-hospital mortality, however, these adverse events are acceptable with a variety of strategies.

## Long-Term Clinical Outcomes in Patients With Prior CABG

The findings from a recent cohort study, comprising 123,780 consecutive PCI procedures from the Pan-London (UK) PCI registry, revealed no significant differences in all-cause mortality between patients with or without previous CABG in propensity-matched population, after both unadjusted and adjusted analyses (74). However, CTO is a special subtype of CAD that represents one of the last barriers in coronary intervention. Currently, data on long-term clinical outcomes in patients with previous exposure to CABG are scarce and unclear. Dautov et al. (53) analyzed a cohort of 470 CTO cases, and found that patients with prior CABG exhibited higher incidences of major adverse cardiac events (MACE) (cardiac death, MI, target-vessel revascularization (TVR), or re-occlusion) (hazards ratio (HR) = 2.2; *p* = 0.02), at 1-year follow up. On the other hand, Azzalini et al. (40) evaluated 2,058 patients who underwent CTO PCI at 7 centers, and found significantly higher 24-month target-vessel failure (cardiac death, target vessel MI, and TVR) rates in patients who had undergone CABG relative to those without prior exposure to CABG (16.1 vs. 9.0%; *p* < 0.001). More recently, Nikolakopoulos et al. (41) analyzed data from the PROGRESS CTO (Prospective Global Registry for the Study of CTO Intervention) registry, and confirmed that, patients with prior CABG exhibited higher incidence of MACE (21.8 vs. 12.7%) and MI, but had similar mortality and repeat revascularization rates after 1 year. Conversely, Toma et al. (58) retrospectively analyzed 2,002 patients who underwent CTO PCI and found that those exposed to CABG presented with a significantly higher risk of 2.6-year MACE (36 vs. 30%, *p* = 0.003), including all-cause death, non-fatal MI, and TVR. However, the authors found no significant differences with regards to MACE (adjusted HR 1. 08, 95% CI 0.86–1.35, *p* = 0.52) after multivariate adjustment. Consistent results were reported in another study, comprising 20,081 patients, from the British Cardiovascular Intervention Society, as evidenced by no significant differences in mortality rates at 1 year (OR 1.02, CI 0.81–1.29, *p* = 0.87) (59). Table 2 outlines the long-term clinical outcomes in patients with prior CABG in major studies.

**Table 2:** Major studies comparing long-term outcomes of pCABG vs. nCABG.

**Study**	**Median follow-up**	**Endpoint**	**Clinical outcomes: pCABG vs. nCABG**
Toma et al. (58)	2.6 years	The primary outcome: all-cause mortality The secondary outcome: MACE (all-cause death, non-fatal MI and TVR)	After multivariable adjustments: all-cause mortality (16 vs. 11%, adjusted HR 1.22, 95% CI 0.86–1.74, *p =* 0.27), MACE (36 vs. 30%, adjusted HR 1.08, 95% CI 0.86–1.35, *p =* 0.52)
Dautov et al. (53)	1 year	MACE (cardiac death, MI, TVR, or target-vessel reocclusion)	Death (4 vs. 1%, *p =* 0.01), MACE (15 vs. 6%, *p =* 0.001)
Azzalini et al. (40)	377 days	TVF (cardiac death, target-vessel MI, and TVR)	2-year outcomes: TVF (16.1 vs. 9.0%, *p* < 0.001), cardiac death (3.8 vs. 1.9%, *p =* 0.02), target-vessel MI (2.0 vs. 0.7%, *p =* 0.04), TVR (11.5 vs. 6.6%, *p =* 0.002)
Nikolakopoulos et al. (41)	110 days	MACE (death, MI, TVR, and coronary revascularization)	1-year outcomes: MACE (21.8% VS. 12.7%, adjusted HR 1.76, 95% CI 1.27–2.45, *p* < 0.001), death (adjusted HR 1.53, 95% CI 0.9–2.6, *p =* 0.1), MI (*p =* 0.04), revascularization (*p =* 0.06)
Shoaib et al. (59)	3.84 years	Mortality and TVR	1-year outcomes: Mortality (3 vs. 2%, *p =* 0.87), TVR (6 vs. 5%, *p =* 0.95)

*CABG, coronary artery bypass grafting; CI, confidence interval(s); CTO, chronic total occlusion; HR, hazard ratio; MACE, major adverse cardiovascular events; MI, myocardial infarction; nCABG, no-CABG; pCABG, post-CABG; TVF, target-vessel failure; TVR, target-vessel revascularization*.

**Figure 2:**
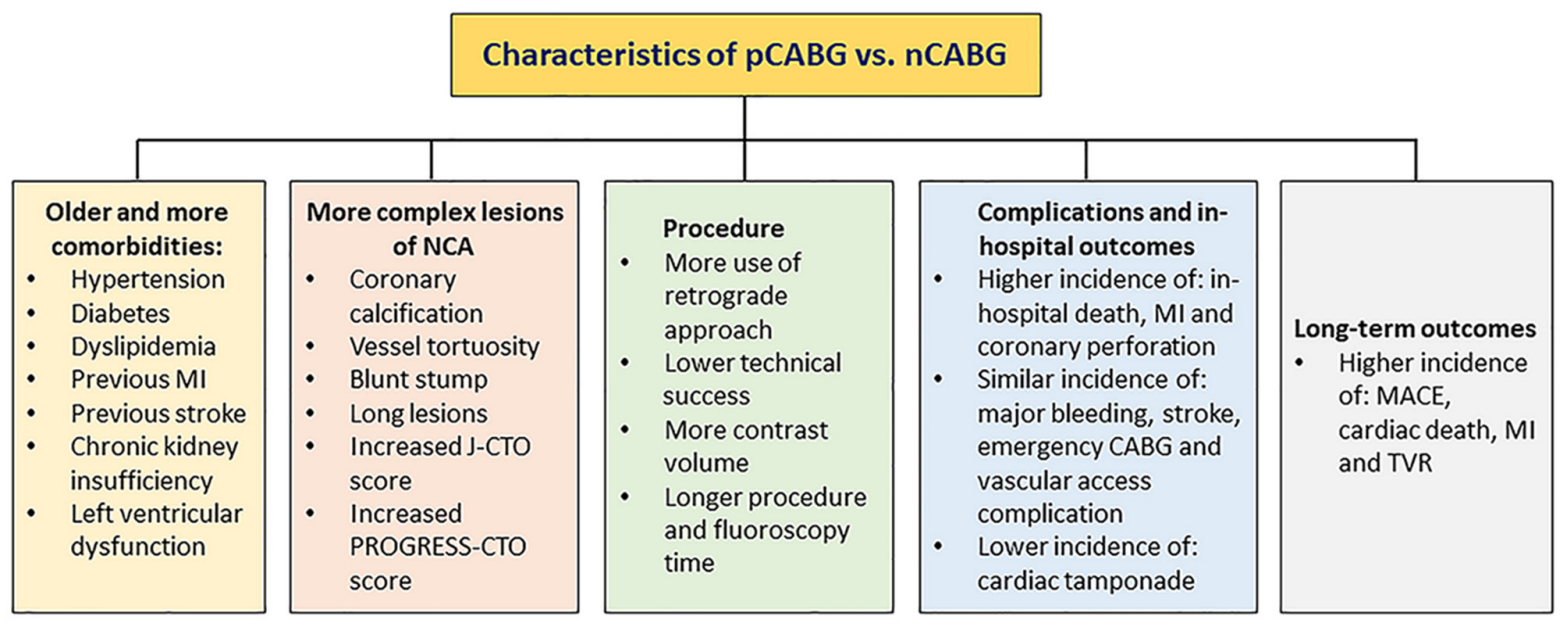
Characteristics of pCABG vs. nCABG. CABG, coronary artery bypass grafting; nCABG, no-CABG; pCABG, post-CABG.

The poor follow-up outcomes of patients with prior exposure to CABG are likely to be related to higher risk baseline coronary anatomy and more comorbidities. Notably, long-term dual antiplatelet therapy (DAPT) is recommended for treatment of patients with prior CABG, owing to the fact that they often have extensive, multilevel atherosclerotic disease and high risk for subsequent adverse cardiovascular events (22, 75). A summary of characteristics for CTO patients with and without prior exposure to CABG is presented in Figure 2.

## Indication for CTO PCI and Objective Benefits

It is important to assess the patient adequately before undertaking the procedure. The decision-making process leading to revascularization for CTOs should pass through three steps: the evaluation of symptoms, the assessment of ischemic burden, and the demonstration of viability (76). In patients with 12.5% or more ischemic myocardium as assessed by myocardial perfusion imaging (MPI) with single photon emission computed tomography (SPECT) and positron emission tomography (PET), revascularization is recommended. Medical therapy is recommended if there is <6.25% ischemic myocardium, as this was associated with increased ischemia at follow-up. In patients with 6.25–12.5% ischemic myocardium, PCI may be reasonable if medical therapy fails to control symptoms (77). In asymptomatic patients who did not have viability data (which were obtained from stress echocardiography, nuclear imaging, magnetic resonance imaging, or PET) available or in subjects with proved absence of viability, medical therapy was strongly preferred. In symptomatic patients, even without information on viability or in asymptomatic patients with viability, PCI was preferred (78). CTO recanalization is indicated in the presence of objective evidence of viability/ischemia in the territory of the occluded artery of more than 10%, as shown by the guidelines on myocardial revascularization (79). Overall, patients with persistent symptoms despite optimized medical therapy and asymptomatic patients with a high burden of ischemia or evidence of viability are suitable candidates for CTO revascularization. Patients who do not fulfill any of these criteria should be managed medically. In addition, clinical and anatomical factors and operator's experience are also important factors which should be taken into consideration during the assessment of a patient candidate for a CTO revascularization.

The 2012 Appropriate use criteria (AUC) guidelines recommend clinicians in making revascularization decisions for their patients with CTO to take into considerations several factors, including patient's symptoms on maximal medical therapy, clinical presentation, risk profile on non-invasive testing, and angiographic features (79). The 2014 European Society of Cardiology/European Association for Cardio-Thoracic Surgery (ESC/EATCS) guidelines for myocardial revascularization also give a Class IIa (B) recommendation, if an ischemia reduction in the CTO territory and/or the relief of angina symptoms can be expected (80). Similarly, the 2011 American College of Cardiology/American Heart Association PCI guidelines state that “PCI of a CTO in patients with appropriate clinical indications and suitable anatomy is reasonable when performed by operators with appropriate expertise” (class IIa, level of evidence B) (32).

The key objectives of CTO recanalization include symptom relief (not only angina), increase in exercise capacity and improvement of quality of life. Several studies have reported that successful CTO revascularization is associated with symptomatic relief of angina, as well as improved left ventricular function, long-term survival, and quality of life (78, 81). A meta-analysis by Hoebers et al. reported that in 34 studies with 2,243 patients, there was a significant improvement in left ventricular ejection fraction (LVEF) by 4.44% following CTO-PCI compared with preintervention LVEF (82). Recently, the Euro-CTO trial showed that, after 12 months follow-up, a greater improvement of Seattle angina questionnaire (SAQ) subscales was observed with PCI as compared with optimal medical therapy (OMT) for angina frequency and quality of life. However, MACEs were comparable between the two groups (83). The DECISION-CTO trial also reported there was no difference in the incidence of MACEs with CTO PCI vs. no CTO-PCI (84).

However, up to now, there is no widely recognized consensus or guideline on treatment strategy of CTO patients with prior CABG, and the prognosis in this population remains controversial. Well-designed, large randomized clinical trials compared PCI with drug eluting stent (DES), optimal medical therapy, and re-CABG for the management of CTO patients with prior CABG are warranted.

## Future Perspectives

Toma et al. (58) reported all-cause mortality (11 vs. 32%; *p* = 0.005) and MACE (31 vs. 50% *p* = 0.01) were significantly reduced in those prior CABG patients with procedural success compared with failed procedure. Due to a higher baseline risk of patients with previous CABG, this afforded a substantially higher absolute reduction in mortality and MACE in patients with previous CABG as compared to that in the non-CABG group. This was consistent with the findings of Iglesias and his colleagues (85, 86), who found that higher-risk patients were highly likely to benefit from the “treatment-risk paradox”, which is a common procedure in PCI. Since patients with prior CABG represent a significant proportion (37%) of those undergoing CTO PCI (87, 88), these findings indicate that this procedure has more clinical benefits in patients with prior compared to those in the non-CABG group. Indeed, those patients with prior CABG are older, with many comorbidities, extensive and complex coronary lesions, and they are more likely to present concurrent cardiovascular risk factors. However, application of the technique and equipment, such as dedicated guidewires, microcatheters and the hybrid approach (especially retrograde approach via SVGs), guarantees high success rates (88–90%) of CTO PCI in patients with prior CABG, and this is accompanied by acceptably low complications rates (39, 58). Overall, this gives us more confidence to apply CTO PCI in these high-risk population, especially in cases where graft intervention or repeat CABG result in unsatisfactory outcomes.

In present era of high success and acceptable complication rates, patient selection for CTO PCI should be focused on those expected to benefit from the procedure, instead of concerns about perceived increased procedural complexity and procedural failure by virtue of prior CABG surgery. Given the higher complexity of CTO PCIs in patients with prior exposure to CABG, these procedures should ideally be performed at experienced centers, by seasoned CTO operators who can promptly treat complications should they arise. Considering the safety and efficacy of these procedures, experienced operators as well as high-volume CTO-PCI centers should focus on high-risk patients. Since approximately half of all patients with prior exposure to CABG have CTO and the patients with SVGs often develop recurrent symptoms, coupled with the high necessity to revascularize CTO patients with prior CABG, we anticipate that this patient population will gain remarkable benefits from this intervention.

## Conclusion

Accordingly, approximately half of all patients with prior exposure to CABG have CTO, and these CTO patients with prior CABG often develop recurrent symptoms and events. Though these patients are with more comorbidities and complex coronary lesions, with the latest refinements equipment and techniques, high success and acceptable complication rates and good prognosis after intervention can be achieved in these patients. CTO PCI in patients with prior CABG is safe and effective when performed in specialized heart teams and by dedicated and experienced CTO operators, and may be actively considered as a treatment option for these high-risk population to achieve complete myocardial revascularization. There is a need for a well-designed and adequately powered sham-controlled, randomized clinical trial to definitively answer the question of the management of CTO patients with prior CABG.

## Author Contributions

LG prepared the manuscript. All authors edited the draft manuscript, and approved the final manuscript.

## Funding

This work was supported by the Natural Science Foundation of Liaoning Province (No. 2020-MS-250).

## Conflict of Interest

The authors declare that the research was conducted in the absence of any commercial or financial relationships that could be construed as a potential conflict of interest.

## Publisher's Note

All claims expressed in this article are solely those of the authors and do not necessarily represent those of their affiliated organizations, or those of the publisher, the editors and the reviewers. Any product that may be evaluated in this article, or claim that may be made by its manufacturer, is not guaranteed or endorsed by the publisher.
